# Study of the influence of psychological mood on the performance and mental health of athletes in VR-aided basketball training

**DOI:** 10.3389/fpsyg.2024.1334111

**Published:** 2024-04-23

**Authors:** Haiyan Wang

**Affiliations:** School of Physical Education, Xinxiang University, Xinxiang, China

**Keywords:** development of basketball in China, gender characteristics of athletes’ performance, mental health of athletes, psychophysiological indicators of athletes, student basketball players, university sports, virtual reality technologies

## Abstract

*The purpose of this study* is to determine the influence of psychological mood on the performance and mental health of athletes during VR training. The study involved representatives of both men’s and women’s basketball teams from universities in China (62 girls and 65 boys, whose average age was 18.2). The participants were divided into 2 groups. Both groups trained regularly, except the experimental group used VR technology, while the control group did not. To study the physical performance of respondents, a complex psychophysiological test was used. The Warwick-Edinburgh Mental Well-Being Scale (WEMWBS) was used to assess the psychological mood and mental health of respondents. The VR training has been proven to increase the psychological attitude of basketball players during the training. Specifically, it has a positive effect on the psychophysiological performance indicators and mental health of athletes. *Prospective research* will be aimed at a comparative study of the impact of VR technology in the training process on the results of basketball players and representatives of other team sports.

## 1 Introduction

Modern education is characterized by the use of virtual learning environments (Shi et al., 2022). Basketball requires an athlete to be tough both physically and mentally, be stress-resistant, and to have the ability to quickly make extraordinary decisions (Shao et al., 2022). The relationship of physical activity with physical and mental health is an interesting topic to study. The importance of the problem of mental health of athletes is evidenced by the increased attention to this issue by the International Society of Sports Psychology. The importance of the athlete’s mental health is emphasized as the main resource that determines the athlete’s sports career and his life after the end of this career (Henriksen et al., 2020). Undoubtedly, physical culture has long been successfully used to treat somatic diseases and mental disorders, such as anxiety and depression. On the other hand, an increase in physical activity may cause the development of psychological abnormalities. Such abnormalities appear as syndromes of somatoform disorders, overtraining, or excessive physical stress (Liu et al., 2018). This is explained by the stressful nature of VR gaming situations (Brand et al., 2013) and underpins the Mental Health Model (MHM) of sports performance (Raglin, 2001). It is assumed that the basketball training system using VR technologies, ensuring an increase in the effectiveness of training, thereby helps to reduce their stress level, having a positive effect on the mental health of athletes (Ma et al., 2020; Tsai et al., 2021). Therefore, the task of ensuring and supporting the psychological well-being of basketball athletes is more relevant than ever, and it is facilitated by the use of VR technology (Richlan et al., 2022).

*The scientific novelty of the study* lies in studying the complex effect of VR technology on the physical endurance and mental health of basketball athletes. Moreover, the study justifies the use of VR technology to guide athletes’ psychological attitude toward achieving successful results.

*The practical significance of the study results* is filed as the possibility of using them to develop Chinese basketball and achieve its leading position in the international arena.

## 2 Literature review

### 2.1 Theoretical justification of the use of VR technologies for training basketball players

In the present day, basketball is an important part of students’ physical education. The use of VR technologies in learning to play basketball is an effective way to foster physical and psychological qualities of athletes (Bedir and Erhan, 2021; Li, 2021). Artificial Intelligence (AI) is seen as an auxiliary technology that can support athletes in the training process (Wei et al., 2021). The use of VR allows to reduce the cognitive anxiety of athletes (Harrison et al., 2021) and helps them learn to control their body, emotions and behavior (Biele, 2022). Ultimately, this leads to better performance (di Fronso et al., 2020) and justifies the importance of gamification both for training athletes (Cmentowski and Krueger, 2021; Uhm et al., 2022) and for solving the sports rehabilitation problems (Yan, 2022). A VR basketball training system allows considering many factors, providing feedback and, thus, improving the efficiency of training (Huang et al., 2019).

### 2.2 The importance of the psychological attitude of basketball players and the impact of VR training on their performance and mental health

The physical and cognitive health of athletes is a necessary factor in achieving sportsmanship and high performance in basketball (Nanda et al., 2021). Some factors such as cohesion, communicative competence, confidence, attention, anxiety, depression, self-esteem and decision making impact athletes’ achievements, and are reflected by the concepts of sports psychology. Additionally, resilience, alertness, and sleep contribute to athletic success, while mental and emotional well-being reduces the risk of injury (Herraro et al., 2020). Mental and physical traumas, as well as stress can cause the rhythm disruption of basketball players’ throws (Meng, 2022). For that reason, it is necessary to identify negative factors in order to prevent mental disorders (Sutcliffe and Greenberger, 2020).

The need to deal with stressful factors requires the development of an early monitoring system that would create strategies for managing stress and preventing its negative consequences (Lopes Dos Santos et al., 2020). The use of Athlete Psychological Stress Questionnaire (APSQ) and the Warwick-Edinburgh Mental Well-being Scale (WEMWBS) is recommended (Stewart-Brown et al., 2009; Rice et al., 2020). During training, it is important to consider each athlete’s personal psychological profile and psychological skills (Obminski et al., 2020), as it has a significant impact on the performance (Nanda and Dimyati, 2019).

### 2.3 The use of VR technology in basketball training

Basketball industry uses AI technologies for training athletes, analyzing and predicting the outcome of competitions, as well as for preventing injuries and increasing enjoyment of the game (Li and Xu, 2021). A highly immersive VR basketball training system allows a comprehensive exploration of the intended structure of natural reality. Hence, it improves the effectiveness of teaching basketball tactics and the overall quality of training (Ma et al., 2020; Jin and Zou, 2021; Tsai et al., 2021).

VR provides a solution to applied tactical and technical problems of basketball teaching and self-learning in accordance with educational standards (Bao and Yao, 2021). The result is the higher technical level of basketball training and the comprehensive development of players’ abilities (Qingtao, 2020; Wei et al., 2022). Furthermore, the use of VR contributes to fundamental changes that ensure progress in the Chinese basketball industry (Le, 2021; Shi, 2021).

Considering the information stated above, it can be concluded that the study of VR technology and its effect on the psychological attitude and mental health of basketball athletes is a relevant topic aimed at increasing the competitiveness of Chinese basketball at the international level (Wang and Song, 2022).

The purpose of the study is to determine the impact of psychological attitude on the performance and mental health of basketball players during VR training.

The objectives of the study are: (1) to analyze data from scientific literature sources regarding the problem of using VR for basketball development; (2) to conduct a psycho-diagnostic study of the impact of psychological attitude on the performance and mental health of basketball players during VR training.

The research hypothesis holds that the use of VR technology when training basketball players helps improve their psychological attitude to the training process, and has a positive effect on the psycho-physiological performance and mental health of athletes.

## 3 Materials and methods

### 3.1 Research design

The study consists of four successive stages. The first stage was to analyze the scientific literature published in the field of basketball development in modern China and the use of VR technology. The impact of online training on the motivation and performance of basketball athletes, their physical and mental health was also analyzed. Although many researchers are attracted to the topic of the psychological health of basketball players who use VR technology, it remains insufficiently studied. Consequently, this very fact determined the purpose and objectives of the study. During the next stage, the hypothesis of the study was formulated, a sample of respondents was created, and valid research methods were identified. The third stage consisted of conducting an empirical study, statistical processing and analyzing the results. The fourth and final stage concluded the study and determined the prospects for further research. The methodological basis of the study was the philosophical understanding of the person’s biopsychosocial nature, the priority role of personal factors in achieving success in sports, as well as understanding The Mental Health Model of sports performance (Raglin, 2001). The said model affirms the presence of a feedback between psychopathology and sports achievements.

### 3.2 Sample

The sample of respondents was determined by the peculiarities of China’s sports policy, focused on basketball development and the search for sports talent through promoting university basketball teams. Both male and female university basketball teams were selected randomly, which made it possible to study the gender aspect of athletes’ online training. The study lasted throughout the whole academic semester. Respondents were divided into 2 study groups: experimental group (32 girls and 33 boys, average age 18.7 years) and control group (30 girls and 32 boys, average age 18.9 years).

During the academic semester, the experimental group was taught using VR technologies, while the control group adhered to the traditional method of learning. In the experimental group, VR technologies were used during the training of basketball students. The VireFit program in basketball game mode was used to practice shooting technique, increase reaction speed, develop accuracy and concentration. Immersive training modes using special VR glasses allowed basketball athletes to analyze the game scenario, which contributed to improving their tactical training.

### 3.3 Study methods

**Figure 1:**
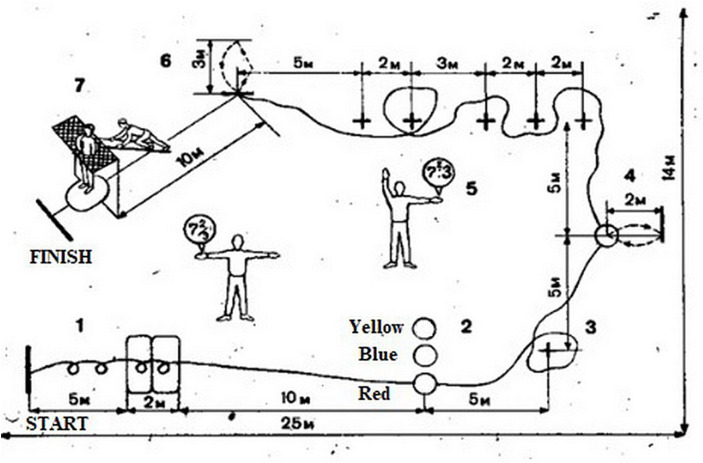
Scheme for performing a complex psychophysiological test.

In order to study the physical performance of the respondents, a complex psychophysiological test developed in 1985 by A. A. Nuzhny and R. N. Makarov was used. Since the test allowed us to assess the important psychophysiological parameters of the respondents, it is valid for use in international sports practice (Burov and Erokhina, 2020). The equipment used for the test included 6 foam-covered mats, 2 blue and 2 red 80 cm racks, 3 basketballs of different colors (red, blue, yellow), one 230 cm high gymnastic crossbar, 1 gymnastic bench, 2 wall targets (1×1 m. each), two stopwatches and 11 nameplates with mathematical examples. The athletes were to run the total distance of 57 m.; the lower edge of the targets was at a height of 2 m (Figure 1).

Test execution algorithm: after hearing the “Go” command, the respondent must perform 2 side flips from a standing position on the start line, then 2 somersaults forward with eyes closed, and then continue running forward. During this time the respondent solves the proposed mathematical example. Depending on the result of the solved example, the respondent chooses a basketball of a certain color (numbers from 5 to 10 represent the blue ball, 15 and more is a red ball, any integer is a yellow ball). After choosing the right ball, the respondent dribbles it around the rack No. 1 and then performs 4 throws at the target No. 1, turning 360° after each throw. Next, the respondent dribbles the ball to rack number 2, and, depending on the results of the next solved mathematical example (obtaining an even or odd number), they pass the ball around the red or blue rack. Target #2 is hit by a ricochet off the floor. Then the respondent gives the ball to the researcher, climbs up the inclined gymnastic bench onto the crossbar and jumps feet down into the hole without touching the edges with their hands. There are 7 checkpoints for capturing the results throughout the entire distance. The criteria for evaluating the test results are presented in Table 1. The respondents are given 3 attempts to complete the test. If the test is passed, the remaining attempts are not used. The evaluation of the stages (in points) is presented in Table 2.

**Table 1:** Criteria for evaluating the complex psychophysiological test results.

No.	Indicators	Checkpoint	External criteria and signs
1.	Vestibular stability	1	Correct body grouping when performing somersaults, taking vertical position, no swaying of the body, rectilinear forward movement
2.	Information perception rate, operational thinking under vestibular irritations	2	Fast digital information reading, correct solution of arithmetic problems
3.	Working memory under vestibular irritations	2	Choosing a ball of the correct color according to the result of the solved arithmetic problem
4.	Movement coordination and accuracy	3	Dribbling and passing around the rack without losing the ball, coordinated work of both hands while dribbling the ball alternately (after passing around the rack)
5.	Fast space orientation under vestibular irritations	4	Precise turns, finding the target and the ball quickly
6.	Ability to extrapolate developments	4	The ball flight time and the time of respondent’s turn around their axis are equal; the respondent does not lose the ball
7.	The task switch ability (distributing and shifting attention between one task and another, performing additional work)	5	Passing the ball around the racks in accordance with the correct solution of the arithmetic problem
8.	Ability to quickly change the activity structure	5	The respondent does not hitch and does not lose the ball when assistant coach gives the command to quickly change the direction of passing the ball around the racks
9.	Retaining fine muscle control during emotional arousal and exhaustion	6	Hitting the ball on the target and catching it after the it bounces
10.	Emotional stability	7	No worrying or fussiness, calm facial expressions and gestures when performing the final exercise
11.	Determination	7	Fast non-stop movement to the crossbar and climbing up the gymnastic bench
12.	Confidence	7	No hitches or delays before jumping into the finish line hole

**Table 2:** Evaluation of athletes’ psycho-physiological qualities on a complex psychophysiological test.

Points	Number of qualities that received a positive mark		
	First attempt	Second attempt	Third attempt
1	-	1–2	1–2
2	1	3	3–4
3	2	4–5	6–8
4	3	6–7	10–12
5	4–5	8–9	-
6	6–7	10–11	-
7	8–9	12	-
8	10–11	-	-
9	12	-	-

Points are given only if the time limit of 70 s is met; otherwise, they are not given at all. The minimum score is 20 and the maximum score is 50.

The psychological attitude and mental health of the respondents were assessed using the Warwick-Edinburgh Mental Well-Being Scale (WEMWBS; Tennant et al., 2007; Stewart-Brown et al., 2009). The methodology scale consists of 14 items that include affective-emotional and cognitive-evaluative aspects and psychological functioning. These items are assessed using a 5-point Likert scale (1 – never, 2 – rarely, 3 – sometimes, 4 – often, 5 – always). The overall minimum score is 14 and the maximum possible score is 70. The higher the score, the higher the level of mental well-being (Tennant et al., 2007).

### 3.4 Data processing

Statistical processing of data was carried out using Microsoft Excel programs (accumulation, sorting, visualization of data) and the Minitab program (analysis of the results obtained). Current calculations were made using the Social Science Statistics online calculator. To determine the statistical reliability and significance of the study results, Student’s t-test was calculated for related and unrelated populations and ANOVA analysis of variance was performed for independent and dependent samples. Cohen’s d test was used to assess the actual difference between scores. The relationship between the studied indicators and scales was assessed using the Pearson correlation coefficient. The significance of VR technology as a factor contributing to improved physical performance and improved mental health stability was assessed based on the χ^2^ criterion, which was calculated using four-field tables.

### 3.5 Ethical issues

Ethical issues were resolved by obtaining permission to conduct the study from the ethics commission and the administrations of the universities. More importantly, the written informed consent to participate in the experiment was obtained from each respondent, ensuring anonymity and confidentiality, academic integrity, and compliance with other bioethical norms. It is worth mentioning that the study did not receive any special funding and there was no conflict of interest. The research was conducted ethically in accordance with the World Medical Association Declaration of Helsinki. The research was approved by the local ethics committees of Xinxiang University (Protocol no. 4993 dated from 02/02/2022). Informed consent was signed by parents of the children.

## 4 Results

Comprehensive psychophysiological testing was carried out twice - at the beginning and at the end of the academic semester. The summarized results of the statistical analysis are presented in Table 3.

**Table 3:** Semester dynamics of psychophysiological indicators of female basketball students of the experimental (A—primary study, A1—repeated study) and control (B—primary study, B1—repeated study) groups.

Experimental group								
Indicators	GPA		Student’s t-test	*p*	ANOVA	*p*	d Cohen	*r*
	A	A1						
1	38.2 ± 0.2	44.3 ± 0.1	301.00	0.00	90600.10	0.00	−39.21	−0.99
2	36.4 ± 0.2	45.1 ± 0.3	275.12	0.00	75690.00	0.00	−34.12	−0.99
3	36.9 ± 0.1	45.2 ± 0.4	86.30	0.00	7447.57	0.00	=28.47	−0.99
4	41.2 ± 0.6	46.4 ± 0.2	41.11	0.00	1690.00	0.00	−11.18	−0.98
5	40.4 ± 0.2	44.1 ± 0.4	58.50	0.00	3422.50	0.00	−11.70	−0.99
6	41.5 ± 0.5	48.2 ± 0.3	105.94	0.00	11222.50	0.00	−16.25	−0.99
7	40.8 ± 0.4	46.1 ± 0.5	142.18	0.00	20216.00	0.00	−11.71	−0.99
8	38.2 ± 0.3	43.8 ± 0.2	138.5	0.00	19182.25	0.00	−21.97	−0.99
9	40.2 ± 0.2	44.5 ± 0.3	135.98	0.00	18490.00	0.00	−16.87	−0.99
10	34.3 ± 0.6	43.0 ± 0.2	67.20	0.00	4515.63	0.00	−19.45	−0.99
11	42.1 ± 0.5	45.1 ± 0.3	44.93	0.00	2018.27	0.00	−7.28	−0.96
12	39.1 ± 0.4	44.8 ± 0.2	90.12	0.00	8122.50	0.00	−18.02	−0.99
Total	39.1 ± 0.3	45.1 ± 0.4	189.74	0.00	35000.00	0.00	−16.97	−0.99
Control group								
Indicators	GPA		Student’s t-test	*p*	ANOVA	*p*	d Cohen	*r*
	B	B1						
1	38.3 ± 0.1	42.0 ± 0.4	47.63	0.00	2268.39	0.00	−12.69	−0.99
2	36.8 ± 0.2	40.5 ± 0.8	19.50	0.00	380.28	0.00	−6.35	−0.95
3	36.5 ± 0.5	41.2 ± 0.3	74.31	0.00	552.50	0.00	−11.40	−0.98
4	41.4 ± 0.2	44.0 ± 0.4	41.11	0.00	1690.00	0.00	=8.22	−0.97
5	40.6 ± 0.1	41.5 ± 0.3	13.81	0.00	190.59	0.00	−4.02	−0.90
6	41.9 ± 0.1	44.0 ± 0.2	59.40	0.00	3528.00	0.00	−13.28	−0.99
7	40.6 ± 0.2	43.9 ± 0.3	104.36	0.00	10890.00	0.00	−12.94	−0.99
8	38.0 ± 0.5	41.2 ± 0.4	101.19	0.00	10240.00	0.00	−7.07	−0.96
9	40.3 ± 0.1	42.2 ± 0.3	29.14	0.00	849.41	0.00	−8.50	−0.97
10	34.6 ± 0.2	35.7 ± 0.5	11.60	0.00	134.44	0.00	−2.89	−0.82
11	42.3 ± 0.1	43.4 ± 0.2	1.62	0.18	2.62	0.18	−6.96	−0.96
12	39.0 ± 0.6	41.1 ± 0.3	22.14	0.00	490.00	0.00	−4.43	−0.91
Total	39.2 ± 0.2	41.7 ± 0.4	39.53	0.00	1562.50	0.00	−7.91	−0.97

1, Vestibular stability; 2, Information perception rate, operational thinking under vestibular irritations; 3, Working memory under vestibular irritations; 4, Movement coordination and accuracy; 5, Fast space orientation under vestibular irritations; 6, Ability to extrapolate developments; 7, The task switch ability (distributing and shifting attention between one task and another, performing additional work); 8, Ability to quickly change the activity structure; 9, Retaining fine muscle control during emotional arousal and exhaustion; 10, Emotional stability; 11, Determination; 12, Confidence.

Initially, the indicators of the experimental and control groups were comparable; there were no statistically or actually significant differences between them (*p* > 0.05; *r* < 0.05). In a repeated study conducted at the end of the academic semester, in both groups—experimental and control—statistically reliable and significant positive psychophysiological dynamics were revealed, which is confirmed by the results of calculating the Student’s test and conducting ANOVA analysis of variance (*p* < 0.05). Cohen’s d (*r* > 0.50) indicates that there is a real difference between the primary and repeat scores in both groups. However, if during the initial study, no statistically and actually significant differences were revealed between the indicators of the experimental and control groups, then the results of the repeated study indicate that the dynamics of the indicators of the experimental group were statistically and actually more significant compared to the control group, as can be seen from Table 4.

**Table 4:** Comparative analysis of the dynamics of indicators of the experimental (A1) and control (B1) groups during repeated research.

Indicators	GPA		Student’s t-test	*p*	ANOVA	*p*	d Cohen	*r*
	A1	В1						
1	44.3 ± 0.1	42.0 ± 0.4	17.03	0.00	289.86	0.00	7.89	0.97
2	45.1 ± 0.3	40.5 ± 0.8	16.80	0.00	282.13	0.00	7.61	0.97
3	45.2 ± 0.4	41.2 ± 0.3	24.34	0.00	592.59	0.00	11.31	0.98
4	46.4 ± 0.2	44.0 ± 0.4	16.18	0.00	261.82	0.00	7.59	0.97
5	44.1 ± 0.4	41.5 ± 0.3	15.82	0.00	250.37	0.00	7.35	0.96
6	48.2 ± 0.3	44.0 ± 0.2	34.29	0.00	1176.00	0.00	16.47	0.99
7	46.1 ± 0.5	43.9 ± 0.3	10.98	0.00	120.62	0.00	5.34	0.94
8	43.8 ± 0.2	41.2 ± 0.4	17.53	0.00	307.27	0.00	8.22	0.97
9	44.5 ± 0.3	42.2 ± 0.3	16.26	0.00	264.50	0.00	7.67	0.98
10	43.0 ± 0.2	35.7 ± 0.5	41.46	0.00	1719.03	0.00	19.17	0.99
11	45.1 ± 0.3	43.4 ± 0.2	13.88	0.00	192.67	0.00	6.67	0.96
12	44.8 ± 0.2	41.1 ± 0.3	30.21	0.00	912.67	0.00	14.51	0.99
Total	45.1 ± 0.4	41.7 ± 0.4	18.44	0.00	340.00	0.00	8.5	0.97

1, Vestibular stability; 2, Information perception rate, operational thinking under vestibular irritations; 3, Working memory under vestibular irritations; 4, Movement coordination and accuracy; 5, Fast space orientation under vestibular irritations; 6, Ability to extrapolate developments; 7, The task switch ability (distributing and shifting attention between one task and another, performing additional work); 8, Ability to quickly change the activity structure; 9, Retaining fine muscle control during emotional arousal and exhaustion; 10, Emotional stability; 11, Determination; 12, Confidence.

As can be seen from this table, as a result of training conducted during the academic semester, the positive dynamics of psychophysiological indicators of student-athletes in the experimental group who used virtual reality technologies was statistically and actually more significant than that of student-athletes in the control group, which is confirmed by the calculation results Student’s t-test (*p* < 0.05), ANOVA analysis of variance (*p* < 0.05), as well as Cohen’s d test (*r* > 0.50).

Further, in the course of the study, the features of the dynamics of physical performance of respondents in the experimental and control groups were analyzed depending on the gender factor. Comparative results of the influence of the training method on the psychophysiological indicators of male student-athletes of the experimental and control groups are presented in Table 5.

**Table 5:** Semester dynamics of psychophysiological indicators of male basketball students in the experimental group (Am—primary study, Am1—repeat study) and the control group (Bm—primary study, Bm1—repeat study).

Indicators	GPA		Student’s t-test	*p*	ANOVA	*p*	d Cohen	*r*
	Am	Am1						
1	40.2 ± 0.2	45.4 ± 0.1	147.08	0.00	21632.00	0.00	−32.89	−0.99
2	38.6 ± 0.4	44.3 ± 0.2	90.12	0.00	8122.50	0.00	−18.02	−0.99
3	38.7 ± 0.3	44.8 ± 0.6	64.30	0.00	4134.44	0.00	−12.86	−0.99
4	42.1 ± 0.3	46.9 ± 0.1	73.63	0.00	5421.18	0.00	−21.47	−0.99
5	41.3 ± 0.2	45.2 ± 0.3	123.33	0.00	15210.00	0.00	−15.30	−0.99
6	40.1 ± 0.3	48.8 ± 0.2	50.55	0.00	2555.16	0.00	−34.12	−0.99
7	41.9 ± 0.1	47.3 ± 0.2	152.74	0.00	23328.00	0.00	−34.15	−0.99
8	36.9 ± 0.4	44.2 ± 0.2	149.41	0.00	22326.00	0.00	−23.08	−0.99
9	39.4 ± 0.6	44.6 ± 0.4	82.22	0.00	6760.00	0.00	−10.19	−0.98
10	38.2 ± 0.4	45.3 ± 0.2	112.26	0.00	12602.50	0.00	−22.45	−0.99
11	44.2 ± 0.3	48.6 ± 0.2	139.14	0.00	19360.00	0.00	−17.26	−0.99
12	44.4 ± 0.1	48.5 ± 0.1	324.13	0.00	105062.50	0.00	−41.00	−0.99
Total	40.5 ± 0.4	46.2 ± 0.2	90.12	0.00	8122.50	0.00	−18.02	−0.99
Indicators	GPA		Student’s t-test	*p*	ANOVA	*p*	d Cohen	*r*
	Bm	Bm1						
1	40.3 ± 0.1	42.2 ± 0.2	53.74	0.00	2888.00	0.00	−12.12	−0.99
2	38.4 ± 0.2	41.4 ± 0.1	84.85	0.00	7200.00	0.00	−18.97	−0.99
3	38.1 ± 0.4	42.5 ± 0.3	139.14	0.00	19360.00	0.00	−12.44	−0.99
4	41.0 ± 0.2	44.8 ± 0.2	4.80	0.00	23.04	0.01	−19.00	−0.99
5	41.6 ± 0.4	42.0 ± 0.2	11.00	0.00	121.00	0.00	−1.26	−0.53
6	40.6 ± 0.2	45.1 ± 0.3	142.30	0.00	20250.00	0.00	−17.65	−0.99
7	41.6 ± 0.4	44.2 ± 0.1	27.03	0.00	730.81	0.00	−8.92	−0.98
8	37.1 ± 0.3	42.0 ± 0.6	51.65	0.00	2667.78	0.00	−10.33	−0.98
9	39.5 ± 0.1	42.0 ± 0.2	70.71	0.00	5000.00	0.00	−15.81	−0.99
10	38.5 ± 0.3	41.2 ± 0.1	41.42	0.00	1715.29	0.00	−12.07	−0.99
11	44.1 ± 0.5	45.0 ± 0.3	14.23	0.00	202.50	0.00	−2.18	−0.74
12	44.6 ± 0.2	45.3 ± 0.2	49.33	0.00	2433.68	0.00	−3.5	−0.87
Total	40.5 ± 0.3	43.1 ± 0.2	82.22	0.00	6760.00	0.00	−10.20	−0.98
Indicators	GPA		Student’s t-test	*p*	ANOVA	*p*	d Cohen	*r*
	Am1	Bm1						
1	45.4 ± 0.1	42.2 ± 0.2	41.26	0.00	1701.98	0.00	20.24	0.99
2	44.3 ± 0.2	41.4 ± 0.1	3.02	0.01	9.11	0.02	18.34	0.99
3	44.8 ± 0.6	42.5 ± 0.3	14.70	0.00	216.00	0.00	4.85	0.92
4	46.9 ± 0.1	44.8 ± 0.2	26.56	0.00	705.60	0.00	13.28	0.99
5	45.2 ± 0.3	42.0 ± 0.2	26.13	0.00	682.67	0.00	2.55	0.99
6	48.8 ± 0.2	45.1 ± 0.3	30.21	0.00	912.67	0.00	12.16	0.98
7	47.3 ± 0.2	44.2 ± 0.1	39.99	0.00	1599.00	0.00	19.61	0.99
8	44.2 ± 0.2	42.0 ± 0.6	10.73	0.00	115.24	0.00	4.92	0.93
9	44.6 ± 0.4	42.0 ± 0.2	16.86	0.00	284.09	0.00	8.22	0.97
10	45.3 ± 0.2	41.2 ± 0.1	52.89	0.00	2797.00	0.00	25.93	0.99
11	48.6 ± 0.2	45.0 ± 0.3	29.39	0.00	864.00	0.00	14.12	0.99
12	48.5 ± 0.1	45.3 ± 0.2	41.28	0.00	1703.83	0.00	20.24	0.99
Total	46.2 ± 0.2	43.1 ± 0.2	32.23	0.00	1038.89	0.00	15.50	0.99

1, Vestibular stability; 2, Information perception rate, operational thinking under vestibular irritations; 3, Working memory under vestibular irritations; 4, Movement coordination and accuracy; 5, Fast space orientation under vestibular irritations; 6, Ability to extrapolate developments; 7, The task switch ability (distributing and shifting attention between one task and another, performing additional work); 8, Ability to quickly change the activity structure; 9, Retaining fine muscle control during emotional arousal and exhaustion; 10, Emotional stability; 11, Determination; 12, Confidence.

As can be seen from this table, in both groups—in the experimental group, where virtual reality technologies were used during the training of student-athletes on basketball teams, and in the control group, where virtual reality technologies were not used during the study—the dynamics of male athletes’ performance was positive. The advantage of VR training is evidenced by the overall average score of male basketball students, which in the experimental group increased from 40.5 ± 0.4 to 46.2 ± 0.2 points, while in the control group it increased from 40.5 ± 0 0.3 to 43.1 ± 0.2 points. This difference between the results of the primary and repeated measurements was significant and significant both statistically (*p* < 0.05) and factually (*r* > 0.50). However, a comparison of the results of a repeated study of the psychophysiological indicators of male basketball students showed that the observed positive semester dynamics were both statistically and actually more pronounced in the experimental group that used virtual reality technologies for training. The statistical reliability and significance of the identified difference is confirmed by the calculation of Student’s t-test (*p* < 0.05), the results of ANOVA analysis of variance (*p* < 0.05) and the values of Cohen’s d test (*r* > 0.50).

Comparison of those presented in Table 5 psychophysiological indicators of male basketball students with data from female basketball players showed that the psychophysiological performance of women is slightly lower than that of men. This fact is explained by anatomical and physiological differences between men and women. At the initial assessment, it was 39.1 ± 0.3 points for female basketball students in the experimental group and 40.5 ± 0.4 points for female basketball students in the control group (*p* > 0.05; *r* < 0.50). In a repeated study, the psychophysiological indicators of female basketball players statistically and actually reliably and significantly increased (*p* < 0.05; *r* > 0.50), amounting to 45.1 ± 0.4 points in the experimental group and 42.8 ± 0.6 in the control group. The gender characteristics of the dynamics of psychophysiological indicators of respondents in the experimental group are evidenced by the results of the study presented in Table 6.

**Table 6:** Comparison of psychophysiological indicators of student basketball teams depending on the gender factor (student basketball players: Am—primary study; Am1—repeated study; female basketball students: Af—primary study, Af1—repeated study).

Indicators	GPA		Student’s t-test	*p*	ANOVA	*p*	d Cohen	*r*
	Am	Af						
1	40.2 ± 0.2	38.2 ± 0.2	23.10	0.00	533.39	0.00	10.00	0.98
2	38.6 ± 0.4	36.4 ± 0.2	14.83	0.00	220.00	0.00	6.96	0.96
3	38.7 ± 0.3	36.9 ± 0.1	1.97	0.04	3.88	0.08	8.05	0.97
4	42.1 ± 0.3	41.2 ± 0.6	2.93	0.01	8.60	0.02	0.00	0.00
5	41.3 ± 0.2	40.4 ± 0.2	8.06	0.00	64.97	0.00	4.5	0.91
6	40.1 ± 0.3	41.5 ± 0.5	−7.38	0.00	54.44	0.00	−3.40	−0.86
7	41.9 ± 0.1	40.8 ± 0.4	8.14	0.00	66.30	0.00	3.77	0.88
8	36.9 ± 0.4	38.2 ± 0.3	−7.91	0.00	62.59	0.00	−3.68	−0.88
9	39.4 ± 0.6	40.2 ± 0.2	−3.90	0.00	15.24	0.00	−1.79	−0.67
10	38.2 ± 0.4	34.3 ± 0.6	16.78	0.00	281.67	0.00	7.65	0.97
11	44.2 ± 0.3	42.1 ± 0.5	10.25	0.00	105.04	0.00	7.00	0.96
12	44.4 ± 0.1	39.1 ± 0.4	29.61	0.00	877.00	0.00	18.18	0.99
Total	40.5 ± 0.4	39.1 ± 0.3	8.52	0.00	72.59	0.00	3.96	0.89
Indicators	GPA		Student’s t-test	*p*	ANOVA	*p*	d Cohen	*r*
	Am1	Af1						
1	45.4 ± 0.1	44.3 ± 0.1	5.57	0.00	30.97	0.00	11.00	0.98
2	44.3 ± 0.2	45.1 ± 0.3	−6.53	0.00	42.67	0.00	−3.14	−0.84
3	44.8 ± 0.6	45.2 ± 0.4	−1.72	0.06	2.96	0.12	−0.78	−0.37
4	46.9 ± 0.1	46.4 ± 0.2	6.32	0.00	40.00	0.00	1.71	0.65
5	45.2 ± 0.3	44.1 ± 0.4	6.69	0.00	44.81	0.00	3.11	0.84
6	48.8 ± 0.2	48.2 ± 0.3	4.90	0.00	24.00	0.00	2.35	0.76
7	47.3 ± 0.2	46.1 ± 0.5	1.14	0.14	1.29	0.29	3.15	0.84
8	44.2 ± 0.2	43.8 ± 0.2	4.40	0.00	19.37	0.00	2.00	0.71
9	44.6 ± 0.4	44.5 ± 0.3	0.60	0.29	0.37	0.56	0.28	0.14
10	45.3 ± 0.2	43.0 ± 0.2	3.24	0.01	4.41	0.07	11.50	0.99
11	48.6 ± 0.2	45.1 ± 0.3	28.58	0.00	816.67	0.00	13.73	0.99
12	48.5 ± 0.1	44.8 ± 0.2	46.80	0.00	2190.40	0.00	23.40	0.99
Total	46.2 ± 0.2	45.1 ± 0.4	7.42	0.00	55.00	0.00	3.48	0.87

1, Vestibular stability; 2, Information perception rate, operational thinking under vestibular irritations; 3, Working memory under vestibular irritations; 4, Movement coordination and accuracy; 5, Fast space orientation under vestibular irritations; 6, Ability to extrapolate developments; 7, The task switch ability (distributing and shifting attention between one task and another, performing additional work); 8, Ability to quickly change the activity structure; 9, Retaining fine muscle control during emotional arousal and exhaustion; 10, Emotional stability; 11, Determination; 12, Confidence.

Most of the psychophysiological indicators of both male and female basketball students during the initial measurement corresponded to the average level. With repeated measurements, in the control group of basketball athletes who trained in the traditional way, psychophysiological indicators statistically and actually reliably and significantly improved (*p* < 0.05; *r* > 0.50), but continued to remain in the range of average values. At the same time, it was revealed that in most psychophysiological parameters, male basketball athletes were superior to female basketball players. This difference was statistically and factually significant and significant (*p* < 0.05; *r* > 0.50). At the same time, female basketball players were superior to male basketball players in the ability to quickly change the structure of activity (38.2 ± 0.3, 38.2 ± 0.3 and 36.9 ± 0.4 points; *p* < 0.05; *r* = −0.88).

Upon repeated research, the level of psychophysiological indicators of both male and female student-athletes increased significantly and reached a range above average. At the same time, female basketball players outperformed male basketball players in psychophysiological indicators related to the functioning of the vestibular apparatus. However, if the indicators of changes in the speed of information perception, operational thinking during vestibular irritations of female basketball players exceeded the similar indicators of male basketball players statistically and in fact reliably and significantly (45.1 ± 0.3 points vs. 44.3 ± 0.2 points, *p* < 0.05; *r* = −0.84), then the statistical and actual difference in working memory indicators during vestibular stimulation in female basketball players and male basketball players was insignificant (45.2 ± 0.4 points vs. 44.8 ± 0.6 points; *p* > 0.05; r = −0.37). VR training resulted in higher development of most psychophysiological qualities in male basketball students than in female basketball students. However, female basketball students had better working memory during vestibular stimulation (45.1 ± 0.3 and 44.8 ± 0.6 points).

Also during this study, the influence of the traditional training method and the training method using virtual reality technologies on the mental health indicators of male and female basketball students was compared. In the next Table 7 shows the results of a study of the semester dynamics of the mental health of male basketball students in the experimental group who used VR technology in training, and male basketball students in the control group who trained without using VR technology during the experimental semester. VR technologies.

**Table 7:** Semester dynamics of mental health indicators of male basketball students in the experimental group (Am—primary study, Am1—re-study), and male basketball students in the control group (Bm—primary study, Bm1—re-study).

Indicators	GPA		Student’s t-test	*p*	ANOVA	*p*	d Cohen	*r*
	Am	Am1						
1	34.4 ± 0.6	44.6 ± 0.4	103.70	0.00	10752.67	0.00	20.00	−0.99
2	35.2 ± 0.3	48.7 ± 0.6	142.30	0.00	20250.00	0.00	−22.5	−0.99
3	26.5 ± 0.3	38.4 ± 0.2	376.31	0.00	141610.00	0.00	−46.68	−0.99
4	30.2 ± 0.4	39.4 ± 0.2	37.16	0.00	1380.63	0.00	−29.09	−0.99
5	40.1 ± 0.3	45.2 ± 0.6	53.76	0.00	2890.00	0.00	−10.75	−0.98
6	35.4 ± 0.2	48.1 ± 0.3	86.00	0.00	7395.16	0.00	−49.81	−0.99
7	33.6 ± 0.4	46.3 ± 0.5	401.61	0.00	161290.00	0.00	−28.05	−0.99
8	34.7 ± 0.3	43.8 ± 0.4	287.77	0.00	82810.00	0.00	−25.74	−0.99
9	31.0 ± 0.2	42.5 ± 0.1	325.27	0.00	105800.00	0.00	−72.73	−0.99
10	33.1 ± 0.5	38.6 ± 0.7	86.96	0.00	7562.50	0.00	−9.04	−0.98
11	36.9 ± 0.1	45.3 ± 0.1	332.04	0.00	110250.00	0.00	−84.00	−0.99
12	42.1 ± 0.3	49.5 ± 0.4	243.01	0.00	54760.00	0.00	−20.93	−0.99
13	43.1 ± 0.2	52.4 ± 0.2	107.59	0.00	11575.12	0.00	−46.50	−0.99
14	38.6 ± 0.4	53.2 ± 0.2	223.95	0.00	50155.29	0.00	−46.17	−0.99
Total	33.2 ± 0.3	45.4 ± 0.4	385.80	0.00	148840.00	0.00	−34.51	−0.99
Indicators	GPA		Student’s t-test	*p*	ANOVA	*p*	d Cohen	*r*
	Bm	Bm1						
1	34.3 ± 0.5	36.3 ± 0.2	20.31	0.00	412.63	0.00	−5.25	−0.93
2	35.4 ± 0.1	38.2 ± 0.4	28.94	0.00	837.61	0.00	−9.60	−0.98
3	26.4 ± 0.6	32.1 ± 0.5	180.25	0.00	32490.00	0.00	−10.32	−0.98
4	30.6 ± 0.2	33.5 ± 0.1	82.02	0.00	6728.00	0.00	−18.34	−0.99
5	41.0 ± 0.4	42.8 ± 0.2	28.46	0.00	810.00	0.00	−5.69	−0.94
6	35.6 ± 0.4	42.3 ± 0.1	70.00	0.00	4900.66	0.00	−22.98	−0.99
7	33.2 ± 0.6	39.2 ± 0.4	43.89	0.00	1926.67	0.00	−11.77	−0.99
8	34.9 ± 0.1	38.2 ± 0.5	25.45	0.00	647.83	0.00	−9.15	−0.98
9	31.1 ± 0.4	35.7 ± 0.5	145.46	0.00	21160.00	0.00	−10.16	−0.98
10	33.4 ± 0.2	36.2 ± 0.4	44.27	0.00	1960.00	0.00	−8.85	−0.98
11	37.1 ± 0.3	40.0 ± 0.2	91.72	0.00	8410.00	0.00	−11.37	−0.98
12	42.2 ± 0.1	44.5 ± 0.3	35.28	0.00	1244.71	0.00	−10.29	−0.98
13	43.4 ± 0.1	48.5 ± 0.1	274.74	0.00	75484.57	0.00	−51.00	−0.99
14	38.8 ± 0.2	42.1 ± 0.5	29.64	0.00	2247.62	0.00	−8.67	−0.97
Total	33.6 ± 0.3	36.2 ± 0.4	129.00	0.00	16641.00	0.00	−7.35	−0.96

1, I have been feeling optimistic about the future; 2, I have been feeling useful; 3, I have been feeling relaxed; 4, I have been feeling interested in other people; 5, I have had energy to spare; 6, I have been dealing with problems well; 7, I have been thinking clearly; 8, I have been feeling good about myself; 9, I have been feeling close to other people; 10, I have been feeling confident; 11, I have been able to make up my own mind about things; 12, I have been feeling loved; 13, I have been interested in new things; 14, I have been feeling cheerful.

Comparative statistical analysis presented in Table 7 shows the advantage of training male basketball students using VR technology compared to traditional training. The positive dynamics of mental health are more pronounced in the experimental group (33.2 ± 0.3 and 45.4 ± 0.4 points, *p* < 0.05, *r* > 0.50) compared to the control group (33.6 ± 0.3 and 36.2 ± 0.4 points, *p* < 0.05, *r* > 0.50).

The results of the study of the dynamics of mental health indicators of female basketball students in the experimental group and female basketball students in the control group are presented in Table 8.

**Table 8:** Comparative dynamics of mental health indicators of female basketball students in the experimental group (Af—primary study, Af1—re-study), and female basketball students in the control group (Bf—primary study, Bf1—re-study).

Indicators	GPA		Student’s t-test	*p*	ANOVA	*p*	d Cohen	*r*
	Af	Af1						
1	34.6 ± 0.2	42.4 ± 0.1	220.62	0.00	48672.00	0.00	−49.33	−0.99
2	32.1 ± 0.3	46.5 ± 0.5	227.68	0.00	51840.00	0.00	−34.93	−0.99
3	28.4 ± 0.6	37.3 ± 0.1	56.01	0.00	3137.03	0.00	−20.69	−0.99
4	33.5 ± 0.3	41.6 ± 0.4	256.14	0.00	65610.00	0.00	−22.91	−0.99
5	38.2 ± 0.4	40.0 ± 0.2	28.46	0.00	810.00	0.00	−5.69	−0.94
6	33.1 ± 0.3	39.3 ± 0.1	120.83	0.00	14599.40	0.00	−27.73	−0.99
7	32.2 ± 0.6	43.2 ± 0.4	40.32	0.00	1625.63	0.00	−21.57	−0.99
8	35.9 ± 0.5	46.0 ± 0.2	126.00	0.00	15876.00	0.00	−26.52	−0.99
9	32.5 ± 0.5	41.4 ± 0.2	93.81	0.00	8801.11	0.00	−23.37	−0.99
10	28.4 ± 0.6	36.9 ± 0.5	268.79	0.00	72250.00	0.00	−15.39	−0.99
11	34.8 ± 0.4	37.1 ± 0.3	72.73	0.00	5290.00	0.00	−6.51	−0.96
12	38.5 ± 0.1	44.3 ± 0.2	183.41	0.00	33640.00	0.00	−36.68	−0.99
13	42.2 ± 0.3	48.6 ± 0.4	202.39	0.00	40960.00	0.00	−18.10	−0.99
14	35.7 ± 0.6	49.2 ± 0.1	84.96	0.00	7217.82	0.00	−31.39	−0.99
Total	34.3 ± 0.4	42.4 ± 0.3	256.14	0.00	65610.00	0.00	−22.91	−0.99
Indicators	GPA		Student’s t-test	*p*	ANOVA	*p*	d Cohen	*r*
	Bf	Bf1						
1	34.8 ± 0.4	35.2 ± 0.5	12.65	0.00	160.00	0.00	−0.88	−0.40
2	31.9 ± 0.1	32.0 ± 0.2	2.71	0.05	7.37	0.05	−0.63	−0.30
3	29.1 ± 0.5	30.2 ± 0.3	17.39	0.00	302.50	0.00	−2.67	−0.80
4	33.8 ± 0.4	35.1 ± 0.3	41.11	0.00	1690.00	0.00	−3.68	−0.88
5	38.4 ± 0.1	38.6 ± 0.2	1.26	0.27	1.60	0.27	−1.26	−0.53
6	33.0 ± 0.5	36.1 ± 0.2	32.68	0.00	1067.78	0.00	−8.14	−0.97
7	32.4 ± 0.2	38.3 ± 0.5	62.19	0.00	3867.78	0.00	−15.49	−0.99
8	36.2 ± 0.3	37.4 ± 0.2	61.00	0.00	3721.00	0.00	−4.71	−0.92
9	32.8 ± 0.2	36.9 ± 0.6	47.72	0.00	22777.05	0.00	−9.17	−0.98
10	29.1 ± 0.4	32.6 ± 0.2	86.50	0.00	7482.25	0.00	−11.07	−0.98
11	34.6 ± 0.5	35.8 ± 0.2	12.65	0.00	160.00	0.00	−3.15	−0.84
12	38.2 ± 0.6	40.0 ± 0.4	28.46	0.00	810.00	0.00	−3.53	−0.87
13	42.4 ± 0.1	44.5 ± 0.2	59.40	0.00	3528.00	0.00	−13.28	−0.99
14	35.9 ± 0.5	38.2 ± 0.3	31.03	0.00	962.67	0.00	−5.58	−0.94
Total	34.5 ± 0.3	36.5 ± 0.3	114.73	0.00	13162.58	0.00	−6.67	−0.96

1, I have been feeling optimistic about the future; 2, I have been feeling useful; 3, I have been feeling relaxed; 4, I have been feeling interested in other people; 5, I have had energy to spare; 6, I have been dealing with problems well; 7, I have been thinking clearly; 8, I have been feeling good about myself; 9, I have been feeling close to other people; 10, I have been feeling confident; 11, I have been able to make up my own mind about things; 12, I have been feeling loved; 13, I have been interested in new things; 14, I have been feeling cheerful.

The mental health indicators of female student basketball players in the primary study were statistically comparable (34.3 ± 0.4 and 34.3 ± 0.4 points, *p* > 0.05, *r* < 0.50). The second study revealed a statistically and actually significant increase in indicators in the experimental group (42.4 ± 0.3 points vs. 36.5 ± 0.3 in the control group, *p* < 0.05, *r* > 0.50; Table 9).

**Table 9:** Comparison of gender characteristics of the dynamics of mental health of basketball students in the experimental group who trained using VR technologies (student basketball players: Am—primary study; Am1—repeated study; female basketball students: Af—primary study, Af1—repeated study).

Indicators	GPA		Student’s t-test	*p*	ANOVA	*p*	d Cohen	*r*
	Am	Af						
1	34.4 ± 0.6	34.6 ± 0.2	−0.98	0.18	0.95	0.36	−0.45	−0.22
2	35.2 ± 0.3	32.1 ± 0.3	10.73	0.00	334.26	0.00	10.33	0.98
3	26.5 ± 0.3	28.4 ± 0.6	−8.76	0.00	76.81	0.00	−4.01	−0.89
4	30.2 ± 0.4	33.5 ± 0.3	−20.08	0.00	403.33	0.00	−9.33	−0.98
5	40.1 ± 0.3	38.2 ± 0.4	11.56	0.00	133.70	0.00	5.37	0.94
6	35.4 ± 0.2	33.1 ± 0.3	20.83	0.00	434.06	0.00	9.02	0.98
7	33.6 ± 0.4	32.2 ± 0.6	6.02	0.00	36.30	0.00	2.75	0.81
8	34.7 ± 0.3	35.9 ± 0.5	−6.32	0.00	40.00	0.00	−2.91	−0.82
9	31.0 ± 0.2	32.5 ± 0.5	−8.52	0.00	72.58	0.00	−3.94	−0.89
10	33.1 ± 0.5	28.4 ± 0.6	18.73	0.00	350.63	0.00	8.51	0.97
11	36.9 ± 0.1	34.8 ± 0.4	15.54	0.00	241.64	0.00	7.20	0.96
12	42.1 ± 0.3	38.5 ± 0.1	23.92	0.00	572.30	0.00	16.10	0.99
13	43.1 ± 0.2	42.2 ± 0.3	7.35	0.00	54.00	0.00	3.53	0.87
14	38.6 ± 0.4	35.7 ± 0.6	12.48	0.00	155.74	0.00	5.69	0.94
Total	33.2 ± 0.3	34.3 ± 0.4	−6.69	0.00	44.81	0.00	−3.11	−0.84
Indicators	GPA		Student’s t-test	*p*	ANOVA	*p*	d Cohen	*r*
	Am1	Af1						
1	44.6 ± 0.4	42.4 ± 0.1	16.29	0.00	265.21	0.00	7.55	0.97
2	48.7 ± 0.6	46.5 ± 0.5	8.77	0.00	76.83	0.00	3.98	0.89
3	38.4 ± 0.2	37.3 ± 0.1	13.91	0.00	193.60	0.00	6.96	0.96
4	39.4 ± 0.2	41.6 ± 0.4	−14.83	0.00	220.00	0.00	−6.96	−0.96
5	45.2 ± 0.6	40.0 ± 0.2	24.30	0.00	540.41	0.00	11.63	0.99
6	48.1 ± 0.3	39.3 ± 0.1	48.79	0.00	2380.49	0.00	39.35	0.99
7	46.3 ± 0.5	43.2 ± 0.4	14.95	0.00	223.49	0.00	6.85	0.96
8	43.8 ± 0.4	46.0 ± 0.2	−14.83	0.00	220.00	0.00	−6.96	−0.96
9	42.5 ± 0.1	41.4 ± 0.2	13.91	0.00	193.60	0.00	6.96	0.96
10	38.6 ± 0.7	36.9 ± 0.5	6.17	0.00	38.03	0.00	2.79	0.81
11	45.3 ± 0.1	37.1 ± 0.3	77.31	0.00	5976.89	0.00	36.67	0.99
12	49.5 ± 0.4	44.3 ± 0.2	35.06	0.00	1229.09	0.00	16.44	0.99
13	52.4 ± 0.2	48.6 ± 0.4	25.62	0.00	656.36	0.00	12.02	0.99
14	53.2 ± 0.2	49.2 ± 0.1	50.60	0.00	2560.00	0.00	25.30	0.99
Total	45.4 ± 0.4	42.4 ± 0.3	18.26	0.00	333.33	0.00	8.49	0.97

1, I have been feeling optimistic about the future; 2, I have been feeling useful; 3, I have been feeling relaxed; 4, I have been feeling interested in other people; 5, I have had energy to spare; 6, I have been dealing with problems well; 7, I have been thinking clearly; 8, I have been feeling good about myself; 9, I have been feeling close to other people; 10, I have been feeling confident; 11, I have been able to make up my own mind about things; 12, I have been feeling loved; 13, I have been interested in new things; 14, I have been feeling cheerful.

The primary study revealed that the mental health indicators of female basketball students are statistically and actually reliably and significantly higher than those of male basketball students (34.3 ± 0.4 and 33.2 ± 0.3 points, *p* < 0.05, *r* > 0.50). Training using VR technologies significantly improved mental health indicators of student-athletes of both sexes (*p* < 0.05, *r* > 0.50). However, the mental health indicators of male basketball students increased more significantly, exceeding the same indicators of female basketball students (45.4 ± 0.4 and 42.4 ± 0.3 points, *p* < 0.05, *r* > 0.50).

A direct close correlation was found between the positive psychophysiological dynamics of athletes and their mental health improvement during VR training (Pearson’s correlation coefficient for female basketball teams is 0.9994, for male teams—0.9976). A chi-square (χ^2^) value in female basketball teams is 6.5971, and in male teams, it equals 5.3455, at *p* < 0.05. When it comes to the mental health of athletes, the χ^2^ value equals 11.1604 and 11.2612 at *p* < 0.05. This confirms the assumption that training using virtual reality technologies helps to increase the performance and mental endurance of male and female basketball athletes. After examining these values, we can conclude that the hypothesis of the study is true.

## 5 Discussion

The development of digital technologies is characterized by their penetration into all spheres of human life. The possibilities of virtual and augmented reality make it possible to create conditions for learning, acquiring and improving the necessary skills. One of the current and promising areas is the use of virtual and augmented reality for training athletes. Despite the fact that the creation of virtual training simulators of sports games is a new direction in the sports industry, their use is becoming increasingly in demand. However, the evidence base for the advantages of VR technologies over traditional training is insufficient, which motivates research in this direction.

One of the factors that determines an athlete’s ability to achieve success is performance, which is based on psychological and psychophysiological mechanisms. The sports environment is characterized by increased stress, which also places increased demands on the mental health of athletes.

The popularity of university sports is growing all over the world. In China, this process is characterized by increased attention to the development of basketball, which is spectacular and contributes to the development of communicative competence, stress resistance and other qualities that make basketball an attractive university sport. The active introduction of VR technologies into the training program of basketball athletes raises the problem of their influence on the psychological and physical state of athletes, their performance and mental health.

The use of innovative information technologies and the positive experiences they entail for basketball players in physical training, stress management and psychological adaptation (Post et al., 2020; Morales Téllez et al., 2021; Talha, 2022) make virtual sports an important part of healthy lifestyle (Bum et al., 2018; Chen and Zhu, 2022; Hamad and Jia, 2022). Moreover, such an approach improves the mental health of athletes (Hurley, 2021), trains their perceptual-cognitive skills (Walton et al., 2018) and optimizes performance (Bertollo and Terry, 2021; Siekańska et al., 2021; Richlan et al., 2022). Scientific literature suggests that students experience equal satisfaction from real-life and virtual basketball training (Bum et al., 2018), but AI-assisted training improves the mental health of athletes to a greater extent than traditional gym training (Zhang, 2022).

The results of our study confirm that basketball training using VR technologies contributes to a more significant improvement in the psychophysiological indicators of athletes than traditional training. Also, the use of VR technologies, as shown by the results of our study, has a positive effect on the mental health of basketball athletes.

In addition, the study revealed features of the dynamics of psychophysical indicators and mental health indicators of basketball students depending on the gender factor. It was shown that most psychophysiological qualities were more developed in male basketball students, but female basketball students had better working memory during vestibular stimulation. In addition, training using VR technologies contributed to improvements in mental health indicators, which were more pronounced in male basketball students.

Thus, the evidence presented in our study shows that VR training has more benefits than traditional training and therefore confirms its effectiveness. The results obtained in the study regarding a direct correlation between psychological factors and athlete injuries coincide with the data of scientific literature sources (Leguizamo et al., 2021). Therefore, there is a need for monitoring and analyzing the psychological attitude of athletes in order to improve their future performance (Lochbaum et al., 2021, 2022; Zhou, 2021). Virtual basketball training is more interactive, visual, targeted and effective (Jiang et al., 2022), which is also confirmed by the results of this study.

### 5.1 Limitations of the study

The limitations of the study include the limited amount of time for conducting the study and a relatively small sample size. These limitations arose because of the peculiarities of forming the university student basketball teams, as well as the complexity of organizing a psychophysiological test. However, due to the randomization procedure and the use of valid research methods, the sample can be considered relevant, and the obtained results are typical for the general population of basketball students.

## 6 Conclusion

To summarize, this study justifies the use of VR technology as a tool to shape a positive attitude among basketball players and help them achieve success. In addition, the impact of VR training on the psychophysiological indicators and mental health was revealed to vary depending on gender. The study also showed the complex effect of VR technology on the physical endurance and mental health of basketball players. VR technologies managed to increase the psychophysiological indicators to an above-average level, which rarely happened during traditional training.

The positive effect of VR training is proven by the overall average psychophysiological score: 46.2 ± 0.2 points for boys and 45.1 ± 0.4 points for girls in the experimental group; 43.1 ± 0.2 and 41.7 ± 0.4 points, respectively, in the control group. The research hypothesis was confirmed. Such a conclusion is extremely valuable for the development of Chinese basketball industry and it is a huge step toward achieving the leading position of Chinese teams in the international arena.

Future research could compare the performance of basketball players and other team sports representatives when using VR technology in the training process.

## Data availability statement

The raw data supporting the conclusions of this article will be made available by the authors, without undue reservation.

## Ethics statement

The studies involving humans were approved by Xinxiang University (Protocol no. 4993 dated from 02/02/2022). The studies were conducted in accordance with the local legislation and institutional requirements. Written informed consent for participation in this study was provided by the participants’ legal guardians/next of kin.

## Author contributions

HW: Writing – review & editing, Writing – original draft, Visualization, Validation, Supervision, Software, Resources, Project administration, Methodology, Investigation, Funding acquisition, Formal analysis, Data curation, Conceptualization.
